# Intraoperative robotic measurements of coronal alignment in total knee arthroplasty correlate with pre‐ and post‐operative long‐leg radiographs

**DOI:** 10.1002/jeo2.70220

**Published:** 2025-03-18

**Authors:** Anoop S. Chandrashekar, Jacob A. Fox, Logan M. Locascio, Gregory G. Polkowski, Martin Faschingbauer, J. Ryan Martin

**Affiliations:** ^1^ Vanderbilt University School of Medicine Nashville Tennessee USA; ^2^ Department of Orthopaedic Surgery Vanderbilt University Medical Center Nashville Tennessee; ^3^ Department for Orthopaedic Surgery Ulm University Ulm Germany

**Keywords:** alignment, artificial intelligence, CORI, knee, robotic total knee arthroplasty

## Abstract

**Purpose:**

This study sought to validate intraoperative robotic measurements of femoral and tibial component coronal alignment in total knee arthroplasty (TKA) by comparing to pre‐ and post‐operative standing, double stance, long‐leg radiographs (LLR).

**Methods:**

This retrospective cohort study included 59 unique patients undergoing primary TKA at a single institution. Pre‐ and post‐operative femoral and tibial coronal alignment were measured on LLRs using a deep learning artificial intelligence model and compared to measurements obtained from the imageless robotic system to evaluate the robot's accuracy and reliability.

**Results:**

Robotic measurements were highly correlated with measurements from preoperative LLR (Pearson *r* ^2^ = 0.68). There was no significant difference in preoperative constitutional alignment between the two methodologies (p = 0.28). Additionally, the intraoperative and post‐operative alignment of femoral and tibial implants were not significantly different (*p* = 0.12 and *p* = 0.95, respectively) and were strongly correlated (Pearson *r* ^2^ = 0.5 and Pearson *r* ^2^ = 0.6 respectively). The mean difference in femoral alignment was 0.43° and the mean difference in tibial alignment was 0.01°.

**Conclusions:**

The findings of this study suggest that there were no significant differences in the coronal alignment of TKA when assessed by a robotic system compared to LLR. This signifies the robotic system's high intraoperative accuracy and reliability in determining coronal alignment.

**Level of Evidence:**

Level III.

**Abbreviations**
ASA: American Society of Anesthesiologists
BMI: body mass index
HKA: hip–knee–ankle
LDFA: lateral distal femur angle
LLR: long‐leg radiograph
MPTA: medial proximal tibia angle
OA: osteoarthritis
SD: standard deviation
TKA: total knee arthroplasty

## INTRODUCTION

Total knee arthroplasty (TKA) is the gold standard treatment for reducing pain and improving functional outcomes in patients with end‐stage osteoarthritis (OA). Despite the great success rates with the surgery and advances in technology since the inception of the procedure, it is estimated that up to 18% of TKAs fail by 25 years [7]. With life expectancy increasing and younger patients undergoing TKA, increasing long‐term implant survivorship is of paramount importance [2]. Due to the increasing number of TKAs performed each year, even a small percentage of error in coronal alignment can put many patients at risk for poor functional outcome, low satisfaction and early failure [4, 11, 17]. Robot‐assisted TKA allows for more accurate implant positioning, thus improving lower extremity alignment and potentially reducing the rate of failure due to mechanical reasons [4, 11, 17]. Accurate implant positioning is critical, as achieving planned coronal alignment post‐operatively is an important determinant of patient outcome.

The CORI Surgical System (Smith‐Nephew) is a handheld imageless total knee system that utilizes a robotic burr and tracker arrays placed on the distal femur and proximal tibia to guide navigation. Previous studies have examined various aspects of the accuracy of this system, including post‐operative alignment in the coronal plane, gap balancing, and surgical outcomes [1, 5, 10]. However, these studies did not assess the accuracy of all measurements obtained by the robot intraoperatively. Thus, validating the accuracy of the surgical system with full‐length hip‐to‐ankle radiographs is a critical need moving forward to ensure appropriate functionality and reliability of the surgical technology.

## METHODS

### Study population

This was a retrospective study of 59 consecutive patients who underwent primary TKA for the indication of OA at a single academic institution. Patients were excluded if they underwent surgery for traumatic, infectious or oncologic indications, lacked the required radiographic data, had undergone prior orthopaedic procedures on the ipsilateral lower extremity, or had undergone TKA without the surgical system of focus. All procedures were performed by a single surgeon (MF). Preoperative and three‐month post‐operative long‐leg radiographs (LLRs) were collected along with basic patient demographics from the medical record (Table 1).

**Table 1:** Patient demographics.

	Patients (*n* = 59)
Age (years, SD)	72.1 (7.6)
Sex (*n*, %Male)	33 (56.7)
BMI (kg/m^2^, SD)	29.5 (4.0)
ASA (µ, SD)	2.5 (0.5)

Abbreviations: ASA, American Society of Anesthesiologists; BMI, body mass index; SD, standard deviation.

### LLR collection

In preparation for potential surgery, patients underwent a pre‐operative weight‐bearing LLR approximately 2–4 weeks before surgery. Imaging was standardized such that the patient's heels were separated by 10 cm, and the patient's patellae were aligned in the anteroposterior projection within the centre of the femoral condyles. This protocol was repeated for the collection of LLR at the three‐month post‐operative timepoint.

### Pre‐ and post‐operative alignment measurement

Pre‐ and post‐operative images were analyzed by a previously developed machine learning model to measure the alignment of the native knee by comparing the mechanical and anatomic axes of the limb [16]. The landmarks utilized by the preoperative machine learning model included the centre of femoral head, the intertrochanteric region, the femoral midshaft, the midpoint of the distal femur, the midpoint of the tibial plateau, the tibial midshaft, and the midpoint of the tibial plafond. An analysis algorithm was applied to the landmark identification model to calculate the difference between the anatomic and mechanical axes at the level of the knee, which was used to determine the overall constitutional alignment of the knee (varus, valgus, or neutral). The machine learning algorithm also utilized these landmarks to calculate the medial proximal tibia angle (MPTA), lateral distal femur angle (LDFA) and hip–knee–ankle (HKA) angle as previously described [13].

Alignment of the femoral and tibial components was determined by the post‐operative machine learning model using landmarks including the femoral head, the intertrochanteric region, the femoral midshaft, the distal aspects of the medial and lateral implant femoral condyle, the medial third and lateral third of the superior border of the tibial base plate, the tibial midshaft and the tibial plafond. An analysis algorithm was applied to the landmark identification model to calculate the overall alignment angles of the final components along with MPTA, LDFA and HKA.

### CORI surgical system

All robotic TKAs in this study were performed using the CORI Surgical System (Smith and Nephew). This semi‐active, imageless system uses intraoperative surface mapping of the patient's anatomy and gap‐balancing evaluation to aid the surgeon in performing accurate bone cuts. All resections were performed with the burr, and they were done at the discretion of the surgeon, who had significant surgical experience with the system. The manufacturer's recommended surgical technique as previously described by Adamska et al. was completed [1]. The ‘preoperative’ measurements obtained were the initial coronal alignment measurements from the system before instrumentation, and the ‘post‐operative’ measurements were the final values obtained from the robot in the operating room.

### Data analysis

A power analysis was conducted for the difference between two means with an estimated effect size of *d* = 0.5 which represents a conservatively low effect size (Cohen's *d*: 0.2–0.5 = low effect size, 0.5–0.8 = medium effect size; >0.8 = large), an alpha = 0.05 and a power level of 0.95. This resulted in a sample size of at least 52 patients with an actual calculated power of 0.97 with the 59 included patients in the study.

The values from the machine learning model measurements were compared to those obtained preoperatively and intraoperatively from the CORI robot using paired *t* tests to assess for significant differences. Pearson correlation coefficients were also calculated to assess the level of correlation. All analyses were conducted within GraphPad Prism (v.10.2.0, GraphPad Software).

### Ethical considerations

All patients provided informed consent for the research use of their data, and the study was approved by our Institutional Review Board (IRB: 231366).

## RESULTS

### Patient demographics

Patient demographic information, including age, sex, body mass index (BMI) and American Society of Anesthesiologists (ASA) score, can be found in Table 1.

### Preoperative alignment accuracy

**Figure 1:**
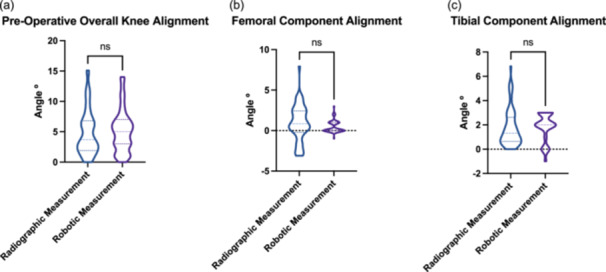
Violin plots. (a) Demonstrating no significant difference in pre‐operative knee alignment between LLR and robot (*p* = 0.28). (b) Demonstrating no significant difference in post‐operative femoral component alignment between LLR and robot (*p* = 0.12). (c) Demonstrating no significant difference in post‐operative tibial component alignment between LLR and robot (*p* = 0.95). LLR, long‐leg radiograph.

**Figure 2:**
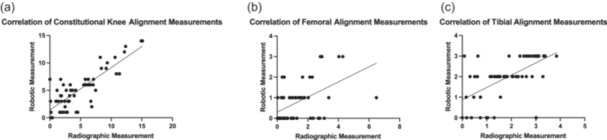
Scatter plots. (a) Demonstrating a strongly positive correlation between the robot and LLR measurements for preoperative knee alignment (*r* ^2^ = 0.68). (b) Demonstrating a moderately positive correlation between the robot and 3‐month post‐operative LLR measurements for femoral component alignment (*r* ^2^ = 0.5). (c) Demonstrating a strongly positive correlation between the robot and 3‐month post‐operative LLR measurements for tibial component alignment (*r* ^2^ = 0.6). LLR, long‐leg radiograph.

A total of 59 patients with preoperative robotic measurement data and preoperative LLR measurements from the deep learning model were assessed to evaluate accuracy of the surgical system. There were no significant differences in the preoperative overall constitutional alignment of the knee on LLR compared with the robotic measurement, with a mean difference of 0.30° (*p* = 0.28, Figure 1). Measurements from the robot and preoperative LLR were highly correlated (Pearson *r^2^* = 0.68) (Figure 2).

### Post‐operative alignment accuracy

Femoral and tibial component alignment measurements from intraoperative robotic data and 3‐month post‐operative LLR were correlated, with the tibia being more positive (Pearson *r* ^2^ = 0.5 and Pearson *r* ^2^ = 0.6, respectively) (Figure 2). At the 3‐month post‐operative time point, there were no significant differences in LLR in the femoral (mean difference 0.43°, *p* = 0.12) or tibial component position (mean difference 0.01°, *p* = 0.95) when compared to the intraoperative robot measurements (Figure 1).

## DISCUSSION

The most important finding of our study was that the intraoperative registration of surface landmarks from the CORI robotic surgical system is highly consistent with measurements obtained from pre‐ and post‐operative imaging. There was a high degree of correlation between the radiographic and robotic measurements taken at the three time points—prior to bony resection (preoperative robotic measurement), at the completion of the procedure but prior to closure (intraoperative robotic measurement), and at the 3‐month follow‐up visit. Therefore, these results should give surgeons confidence that the readings received during the surgery from the device are reasonably accurate and will be within the desired parameters during subsequent follow‐up imaging.

There are several previous studies that assess the surgical performance and post‐operative outcomes following the usage of this robotic system, but none of the prior studies assess the validity of all aspects of the radiographic output [1, 5, 10]. Compared to these previous studies, we further validated the measurements of the tibial component, showing high accuracy with the positioning of the implant. Further, it is important to discuss that preoperative and intraoperative measurements from the robot are non‐weight‐bearing and still highly correlated to weight‐bearing LLR pre‐ and post‐operatively. This is a meaningful and interesting finding, as weight‐bearing is believed to influence the coronal alignment of LLR [18]. Indeed, Colyn et al. performed a systematic review looking at weight‐bearing versus supine HKA and found an overall difference in HKA between supine and weight‐bearing standing films to be 1.76° (*p* < 0.0001) [6, 8, 14, 15]. Finally, in addition to the differences in alignment that weight‐bearing may cause, it has also been reported that there can be up to 4° of variation with alignment measures due to knee external/internal rotation and flexion [3, 9, 12]. In stark contrast to our results, these previously reported findings would indicate that the robotic measurements should not correlate well with LLR pre‐ and post‐operatively. This is a topic that begs further study to understand why the robotic‐assisted systems at times provide measurements that mimic weight‐bearing imaging and other times do not.

There are a few limitations in our study which need to be mentioned. First, all surgeries were performed by an experienced surgeon at a single institution. Further multicenter research would allow for findings that are more representative of the general population. Another limitation is that the LLRs taken were by different radiology technicians. Although standardized protocols were followed, this may still affect the alignment as measured using these radiographs. Additionally, this study was retrospective, which has inherent flaws and is associated with biases that prospective studies eliminate. Finally, although we compared the intraoperative measurements to post‐operative LLR, computerized tomography scans could be a more accurate way of determining the accuracy of the intraoperative measurements in the coronal plane.

## CONCLUSIONS

The findings of this study suggest that imageless surface‐based registration using the CORI robotic system is as accurate as image‐based registration.

## AUTHOR CONTRIBUTIONS

The following authors, Anoop S. Chandrashekar, Jacob A. Fox, Logan M. Locascio, Gregory G. Polkowski, Martin Faschingbauer and J. Ryan Martin, have participated in the content and design of the study and have seen and agreed with the contents of the manuscript.

## CONFLICT OF INTEREST STATEMENT

The authors declare no conflict of interest.

## ETHICS STATEMENT

Institutional review board approval (#231366) was obtained for this study.

## Data Availability

All data are available upon reasonable request with formal authorization from the hospital.
